# Prenatal Phenotype in a Neonate with Prader–Willi Syndrome and Literature Review

**DOI:** 10.3390/diagnostics15131666

**Published:** 2025-06-30

**Authors:** Libing Luo, Mary Hoi Yin Tang, Shengmou Lin, Anita Sik-Yau Kan, Cindy Ka Yee Cheung, Xiaoying Dai, Ting Zeng, Yanyan Li, Lilu Nong, Haibo Huang, Chunchun Chen, Yue Xu, Kelvin Yuen Kwong Chan

**Affiliations:** 1 Prenatal Diagnosis Centre, The University of Hong Kong-Shenzhen Hospital, Shenzhen 518053, China; 2 Shenzhen Clinical Research Center for Rare Diseases, Shenzhen 518053, China; 3 Department of Obstetrics and Gynaecology, Queen Mary Hospital, The University of Hong Kong, Hong Kong 999077, China; 4 Department of Ultrasound, The University of Hong Kong-Shenzhen Hospital, Shenzhen 518053, China; 5 Neonatal Intensive Care Unit, The University of Hong Kong-Shenzhen Hospital, Shenzhen 518053, China; 6 Department of Obstetrics and Gynaecology, The University of Hong Kong-Shenzhen Hospital, Shenzhen 518053, China; 7 School of Nursing and Health Sciences, Hong Kong Metropolitan University, Hong Kong 999077, China

**Keywords:** Prader–Willi syndrome (PWS), prenatal, phenotype, ultrasound

## Abstract

**Background and Clinical Significance:** Prader–Willi syndrome (PWS) is a rare genetic disease caused by imprinted gene dysfunction, typically involving deletion of the chromosome 15q11.2-q13 region, balanced translocation, or related gene mutations in this region. PWS presents with complex and varied clinical manifestations. Abnormalities can be observed from the fetal stage and change with age, resulting in growth, developmental, and metabolic issues throughout different life stages. **Case Presentation:** We report the prenatal characteristics observed from the second to third trimester of pregnancy in a neonate with PWS. Prenatal ultrasound findings included a single umbilical artery, poor abdominal circumference growth from 26 weeks, normal head circumference and femur length growth, increased amniotic fluid volume after 30 weeks, undescended fetal testicles in the third trimester, small kidneys, and reduced fetal movement. The male infant was born at 38 weeks of gestation with a birth weight of 2580 g. He had a weak cry; severe hypotonia; small eyelid clefts; bilateral cryptorchidism; low responsiveness to medical procedures such as blood drawing; and poor sucking, necessitating tube feeding. Blood methylation-specific multiple ligation-dependent probe amplification (MS-MLPA) showed paternal deletion PWS. Notably, this case revealed two previously unreported prenatal features in PWS: a single umbilical artery and small kidneys. **Conclusions:** Through literature review and our case presentation, we suggest that a combination of specific sonographic features, including these newly identified markers, may aid clinicians in the early diagnosis of PWS.

## 1. Introduction

Prader–Willi syndrome (PWS), first described in 1956 by Prader, Labhart, and Willi, is characterized by lifelong, unremitting hyperphagia with accompanying endocrine disorders, cognitive deficits, sensory abnormalities, and behavioral difficulties [1]. It is one of the first confirmed genetic disorders involving genomic imprinting. The prevalence of PWS ranges from 1 in 10,000 to 1 in 30,000 across different populations [2,3,4], with most cases being sporadic. The pathogenesis of PWS is absence of paternal gene expression in the 15q11.2-q13 region, which can arise from paternal chromosome microdeletion, maternal uniparental disomy, or defects in the imprinting center [3,4,5,6,7]. The pathogenic cause and genetic mechanism of PWS are complex, and the vast majority of cases are de novo. PWS can be more readily identified by postnatal features including severe hypotonia; feeding difficulties with sucking deficit; and characteristic facial traits such as a narrow forehead, almond-shaped eyes, a narrow nasal bridge, a thin upper lip, and downturned mouth corners. These features tend to become more pronounced over time. The clinical manifestations of PWS vary greatly with age. Low muscle tone and inability to suckle can lead to feeding difficulties in infancy; bulimia beginning in childhood can lead to obesity; developmental retardation, short stature, cognitive impairment, gonadal dysplasia, and behavioral problems may appear with age [6,7,8,9,10,11]. Obesity and its complications are the leading causes of death among patients with PWS.

Prenatal diagnosis of PWS is challenging due to the lack of known specific prenatal phenotypes. Some cases of prenatally diagnosed PWS were identified through whole-genome noninvasive prenatal screening (NIPS), followed by confirmatory invasive testing via amniocentesis [12,13]. Few studies have reported the antenatal clinical features of fetuses with PWS, including polyhydramnios, reduced fetal movement, and abnormal fetal presentation, which are not unique to PWS [14,15,16,17,18,19]. This report presents a case of fetal PWS, showcasing changing prenatal ultrasound findings between the second and third trimesters. Notable findings include a single umbilical artery, poor growth of abdominal circumference, polyhydramnios, cryptorchidism, slightly smaller kidneys, and reduced fetal movement.

## 2. Case Presentation

A healthy 37-year-old multipara was referred to the Prenatal Diagnosis Center of HKU-SZH due to the detection of a single umbilical artery at 23 weeks of gestation in March 2022. She had previously given birth to a healthy 3.5 kg male newborn at term by cesarean section in 2011. The patient’s medical and family history were unremarkable. The current pregnancy was a natural conception. At 13 weeks of gestation, nuchal translucency thickness was 1.1 mm, crown-rump length was 70 mm, and first-trimester Down syndrome combined screening and non-invasive prenatal testing (NIPT) indicated low risk. An ultrasound scan at 23 weeks revealed an estimated fetal weight (EFW) of 590 g (47th percentile), an abdominal circumference (AC) of 184 mm (51st percentile), a single umbilical artery (SUA), normal fetal morphology, and normal amniotic fluid volume.

**Figure 1:**
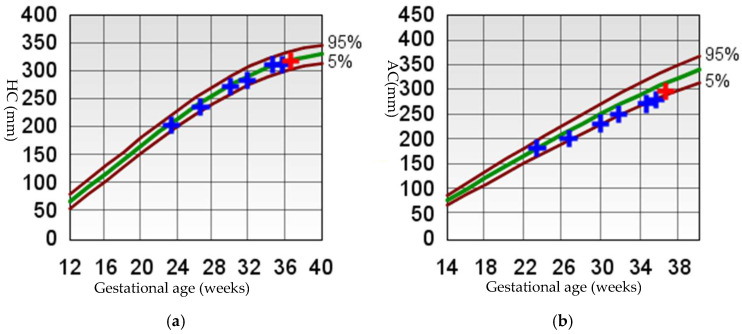

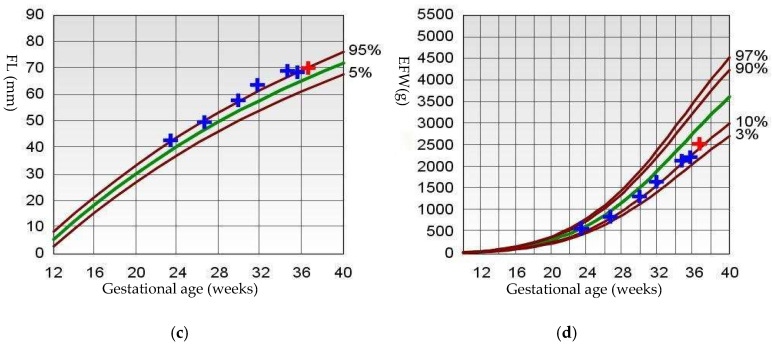
Fetal growth curve [24,25]. (**a**) HC, head circumference; (**b**) AC, abdomen circumference; (**c**) FL, femur length; (**d**) EFW, estimated fetal weight. For (**a**–**c**) The brown curves represent the 5th and 95th percentile lines, and the green curve represents the 50th percentile line. For (**d**) The brown curves represent the 3rd, 10th, 90th, and 97th percentile lines, and the green curve represents the 50th percentile line. The crosses indicate the values from each ultrasound measurement, with blue crosses representing previous measurements and red crosses representing the last ultrasound measurement.

**Figure 2:**
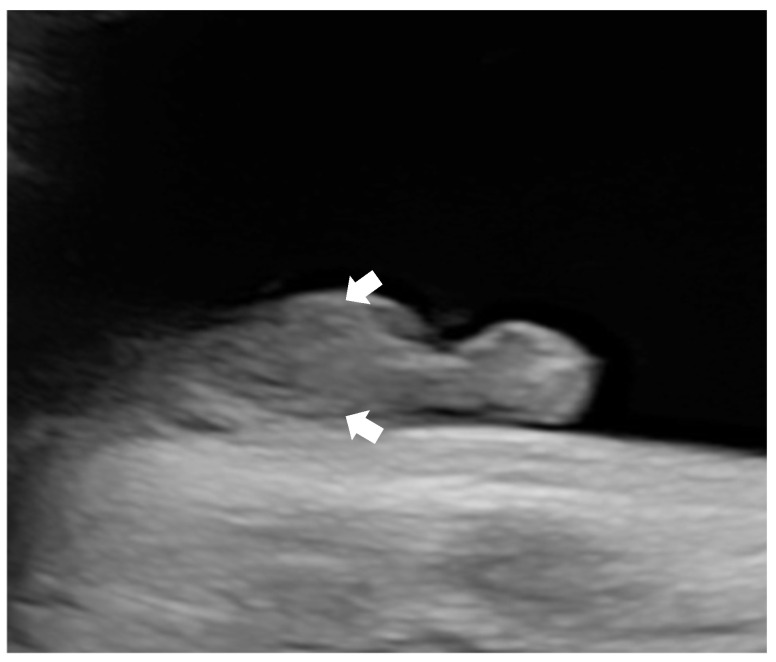
Ultrasonographic image of bilateral crytorchidism (arrows).

**Figure 3:**
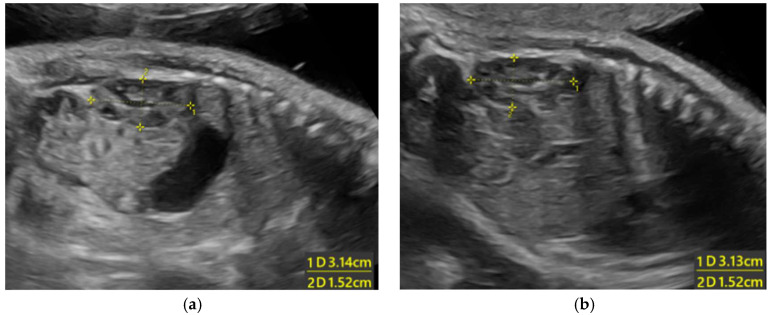
Ultrasonographic images of small kidneys (length of the kidneys are below the 3rd percentile for 34 weeks weeks) [20]: (**a**) left kidney; (**b**) right kidney.

Subsequent ultrasounds showed slow growth of the abdominal circumference with normal head circumference and femur length growth (Figure 1), as well as a gradual increase in amniotic fluid volume. At 26 weeks, EFW and AC were, respectively, 860 g (15th percentile) and 204 mm (9th percentile), with normal amniotic fluid volume. By 29 weeks and 6 days, EFW and AC were, respectively, 1320 g (15th percentile) and 234 mm (7th percentile), with mild polyhydramnios (single deepest pocket: 96 mm; amniotic fluid index [AFI] of 233 mm). At 34 weeks and 4 days of gestation, EFW and AC were, respectively, 2160 g (15th percentile) and 273 mm (4th percentile), with bilateral cryptorchidism (Figure 2) and slightly smaller kidneys (both measuring 31 × 15 mm, below the third percentile) (Figure 3) [20,21,22]. Moderate polyhydramnios was also observed (single deepest pocket: 127 mm; AFI: 376 mm). Fetal movement was minimal during each ultrasound scan, characterized by only a few general movements of the head, trunk, and extremities, with no distinctive sequencing of the body parts and with variance in participating body parts (so-called complexity [23]). This was noted even after additional stimulation, such as pushing on the maternal abdomen or using sound. However, the pregnant woman consistently reported feeling significant fetal movement.

**Figure 4:**
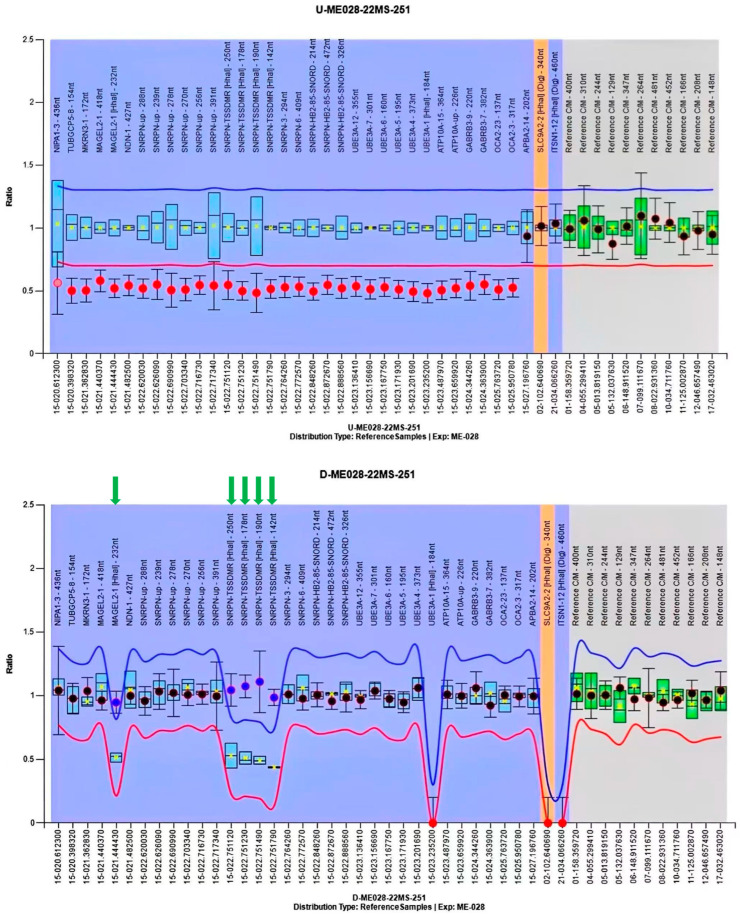
Methylation-specific multiplex ligation-dependent probe amplification (MS-MLPA) result of the proband. The upper panel presents the copy number analysis, while the lower panel illustrates the methylation status analysis. In the upper panel, the ratio of the copy number probes (indicated by red dots) of the undigested DNA of the proband was ~0.5, indicating presence of a single copy number (heterozygous deletion). In the lower panel, the ratio of the methylation-specific probes (denoted by green arrows) of the digested DNA of the proband was ~1.0, meaning that the single copy number region was not digested by the methylation-sensitive restriction enzyme *Hha*I; this implies that the single copy region is methylated. The MS-MLPA findings confirmed the diagnosis of a paternal deletion of PWS.

Given the emerging ultrasound features, including a consistently small abdominal circumference, progressive polyhydramnios, bilateral cryptorchidism, small kidneys, and persistently reduced fetal movement, amniocentesis was recommended for genetic study. In China, there are no legal restrictions on termination of pregnancy due to fetal abnormalities, even in late gestation, provided that the diagnosis is confirmed and the parents are fully informed of the potential outcomes. However, the couple declined amniocentesis. A multidisciplinary team discussed the potential perinatal complications associated with a possible diagnosis of PWS in the fetus, and a perinatal management plan was developed at 36 weeks and 1 day of gestation that included strategies for preventing and treating delivery-related complications, managing possible neonatal issues such as asphyxia and poor sucking, and providing counseling about genetic testing for early diagnosis, as well as considerations for short- and long-term complications and follow-up. A 2580 g male newborn was delivered at 38 weeks and 1 day of gestation by Cesarean section because of previous Cesarean section. The newborn displayed several concerning features, including a small eyelid cleft, a weak cry, slightly low muscle tone, bilateral cryptorchidism, and a diminished response to medical procedures like blood draws, and tube feeding was necessary due to poor sucking. Ultrasound showed slightly small kidneys. Methylation-specific multiplex ligation-dependent probe amplification (MS-MLPA) for the Prader–Willi/Angelman on 15q11-q13 region (SALSA MLPA Probemix ME028, MRC-Holland) was performed, according to the manufacturer’s instructions, on the genomic DNA extracted from the peripheral blood of the patient. The result showed a single copy loss (or heterozygous deletion) at the 15q11.2-q13.1 chromosomal region, which was shown to be methylated, suggesting a paternal deletion of PWS (Figure 4). A lactation specialist instructed breastfeeding function exercise during hospitalization. Before discharge, a long-term management plan was created and discussed with the parents, which included an endocrine assessment, lactation support, and pediatric surgical follow-up.

## 3. Literature Review

A literature search was conducted using PubMed with the keywords “fetal and PWS”, “prenatal and PWS”, and “PWS and fetal ultrasound” from the database’s inception until June 2024. Table 1 summarizes the prenatal characteristics associated with PWS. Most common prenatal manifestations of PWS include decreased fetal movements, polyhydramnios, and fetal growth restriction (FGR). FGR [7,16,26,27,28,29,30,31,32,33], typically asymmetrical, was observed in 21.1% to 65% of cases, characterized by an increased head-to-abdomen circumference ratio or decreased abdominal circumference [26,27,30,31,33]. Decreased fetal movements were reported in 27% to 92% of cases [7,16,26,27,28,29,30,31,32,33]. Polyhydramnios was present in 23% to 46% of cases [7,16,26,27,28,29,30,31,32,33]. Breech presentation was reported in 25.7% to 63.6% of fetuses [16,26,27,28,29,30,31], and cryptorchidism was observed in 87% to 96.7% of male fetuses [26,29,30]. Unusual hand and foot positioning was noted in 9.1% to 25.7% of cases [16,29,32,34,35]. Other less common findings including enlarged lateral ventricles [34,35,36], non-reactive cardiotocography (CTG) pattern [14,31], fetal cardiac rhabdomyoma [33], large biparietal diameter [14], and hydronephrosis [26] have also been seen in different individual cases. Small kidneys and single umbilical artery (SUA) have not been previously reported in PWS cases.

## 4. Discussion

PWS presents with complex and varied clinical manifestations, with abnormalities emerging in the fetal stage and evolving with age. This progression leads to multisystem complications, including respiratory and sleep disorders (e.g., obstructive sleep apnea, obesity hypoventilation syndrome), endocrine and metabolic dysfunction (e.g., adrenal insufficiency, diabetes), and orthopedic issues (e.g., scoliosis, hip dysplasia). Behavioral challenges such as hyperphagia and psychiatric comorbidities further complicate management. The syndrome carries a high mortality rate due to respiratory failure, sudden death during sleep, and severe infections, culminating in a poor prognosis [36,37,38,39,40,41,42,43]. Instead of curative treatment, patients receive symptomatic care. Without early diagnosis and care, patients often experience excessive weight gain [44]. Various nutritional problem cases have been documented, often resulting in severe obesity that usually begins between the ages of 3 and 4 [9,10].

Early diagnosis facilitates parental counseling, helps anticipate and prevent obstetric complications during labor, and ensures better preparation for neonatal care. Comprehensive multidisciplinary care is essential for improving outcomes [45,46,47]. This offers parents guidance and support to prevent aspiration and asphyxia in infancy, reduce obesity later in childhood, and stimulate cognitive and adaptive skills, thereby enhancing the patient’s quality of life [48]. This approach can significantly reduce the severe short- and long-term effects of PWS.

PWS arises from the loss of expression of paternally inherited genes in the 15q11.2-q13 chromosomal region. About 70% of PWS cases are caused by errors in genomic imprinting resulting from a paternal deletion, while maternal uniparental disomy accounts for roughly 25% of cases. Fewer cases stem from defects in the imprinting center, such as microdeletions or epimutations on chromosome 15. Methylation-specific multiplex ligation-dependent probe amplification (MS-MLPA) can diagnose PWS by detecting maternal-only imprinting at 15q11.2-q13. It can also identify the size of the paternally inherited deletion in this region, as well as detect deletions in the imprinting center and SNORD116 [7,17,47,49]. Therefore, MS-MLPA should be the preferred diagnostic method for PWS. While MS-MLPA cannot differentiate between uniparental disomy (UPD) 15 and an imprinting defect caused by epimutation, this limitation only impacts the assessment of recurrence risk and not the patient’s diagnosis [19,50].

However, the lack of a clear fetal phenotype for PWS and limited awareness of it has resulted in very few prenatal diagnoses, which mostly occur accidentally through cytogenetic molecular techniques. In a study of 38 children with PWS, none received a prenatal diagnosis, even though 9 underwent invasive tests that returned normal karyotypes; however, specific genetic tests for PWS were not conducted [27]. Therefore, understanding the fetal features of PWS is crucial for facilitating requests for specific prenatal genetic tests for diagnosis.

While prenatal symptoms cannot guarantee a definitive diagnosis, they can assist clinicians in diagnosing PWS and should prompt early parental counseling and careful decision making during pregnancy and delivery. The literature review identifies decreased fetal movements, FGR, and polyhydramnios as the most common prenatal features of PWS. FGR typically presents as asymmetrical, which can be identified by a higher head-to-abdomen circumference ratio or a smaller abdominal circumference. Among male fetuses, the prevalence of cryptorchidism ranges from 87% to 96.7% [26,29,30]. Breech presentation occurs in 25.7% to 63.6% of fetuses, likely due to increased movement from excessive amniotic fluid. Polyhydramnios is a common finding in fetuses with congenital neuromuscular diseases, probably caused by the weakness of swallowing movements, in analogy with the sucking problems encountered in neonatal life [14]. Additionally, abnormal extremities were noted in 9.1% to 25.7% of cases, possibly related to fetal central nervous system disease [16,29]. It is unclear whether certain uncommon ultrasound findings—such as enlarged lateral ventricles [26,34,35], non-reactive CTG patterns [14,31], fetal cardiac rhabdomyoma [33], large biparietal diameter [14], and hydronephrosis [26]—are characteristic of PWS. Further studies are necessary to identify specific prenatal phenotypes associated with PWS.

Our case illustrates the characteristics of a PWS fetus from the middle to the late stages of pregnancy. We noted that the clinical features mainly present after 26 weeks of gestation, similar with previous reports [29]. Regarding estimated weight, although the fetus did not meet the diagnostic criteria for FGR, poor growth in abdominal circumference aligns with literature findings. It shows that PWS is often associated with asymmetrical FGR, which is characterized by either an increased head-to-abdomen circumference ratio or a decreased abdominal circumference. A small abdominal circumference may be a more significant characteristic of PWS than reduced fetal weight. In our case, the fetal movements observed with ultrasound were inconsistent with those reported by the pregnant woman, suggesting that in the case of polyhydramnios combined with FGR, attention should be paid to the observation of fetal movement during ultrasound scanning, rather than relying on maternal perception which is subjective. While the presence of a single umbilical artery in our PWS fetus could be coincidental, conducting thorough and regular ultrasound assessments of fetuses with soft markers is crucial. This approach may enhance the chances of discovering new clinical clues for early disease diagnosis. In our case, smaller kidneys were first noted on ultrasound examination. Because measuring fetal kidneys is not standard in routine ultrasound examinations, we cannot determine if smaller kidneys are common in PWS fetuses or merely coincidental. However, it is reasonable to consider a potential association, and documenting this combination may aid future investigations into the genotype–phenotype correlation of PWS. To validate the inclusion of a single umbilical artery and small kidneys as markers for PWS in prenatal scans, a large sample size study is needed to confirm their consistency across PWS fetuses.

The complex pathogenesis of PWS, combined with the limitations of genetic tests, means that relying on a single test may result in missed diagnoses. For instance, G-banding does not detect chromosomal microdeletions, maternal uniparental disomy, or defects in the imprinting center, while chromosomal microarray fails to identify maternal uniparental disomy or point mutations [33,35,46]. Antenatal diagnosis of PWS can be achieved using specific molecular genetic tests, such as methylation-specific multiplex ligation-dependent probe amplification (MS-MLPA). Understanding the prenatal features of PWS allows for a heightened suspicion of the disorder, which can facilitate early diagnosis through targeted genetic testing. Notably, fetal growth restriction—a key prenatal feature of PWS—exemplifies how epigenetic dysregulation (e.g., aberrant methylation at 15q11.2-q13) can alter developmental programming. As highlighted by Salmeri et al. [51], elucidating the role of epigenetics in the developmental origins of health and disease represents a new challenge for the coming years, with the goal of providing early interventions and prevention strategies and, hopefully, new treatment opportunities. In cases without invasive prenatal diagnosis, the fetal condition may be suspected before birth, enabling the family to prepare for a potential diagnosis. Multidisciplinary intervention could be started early, and newborns can receive the attention of serial multidisciplinary team assessment for the earliest diagnosis and management to improve outcome in early and later life.

## 5. Conclusions

PWS should be listed in differential diagnoses if a fetus has the following features: polyhydramnios, decreased fetal movements, and poor growth of abdominal circumference. Further comprehensive serial ultrasound assessment is needed to see if additional possible features of PWS are present. This report identifies the presence of small kidneys and a single umbilical artery as potential new prenatal sonographic markers for PWS. Understanding more of the prenatal characteristics of PWS helps to select effective diagnostic tests in the prenatal period to avoid missed diagnosis.

## Figures and Tables

**Table 1:** Summary of prenatal features of PWS.

	Cohort						Case Report							
	Geysenbergh [16]	Bar [27]	Gross [28]	Zhou [29]	Grootjen [30]	Srebnik [26]	Bigi [34]		L’Herminé [35]	Murata [31]	Dong [32]	Traisrisilp [33]	Hiroi [14]	Our Case
							Case 1	Case 2						
Number of Patients	11	61	37	226	244	192								
DFM	10/11 (90.9%)	15/56 (27%)	34/37 (92%)	202/226 (89.4%)	78.5%	80/101 (80.4%)	+	+	N.R.	-	+	+	-	+
FGR	7/11 (63.6%)	17/56 (30%)	24/37 (65%)	100 (44.2%)	50/244 (21.1%)	57/101 (57.6%)	+	-	N.R.	-	+	+	+	-
Increased H/A ratio	N.R.	N.R.	23/37 (62%)	N.R.	N.R.	30/67 (44.8%)	N.R.	+	N.R.	-	+	N.R.	+	+
Decreased AC	N.R.	N.R.	N.R.	N.R.	N.R.	56/64 (87.5%)	+	+	N.R.	-	+	N.R.	+	+
Breech position	7/11 (63.6%)	N.R.	14/37 (38%)	58/226 (25.7%)	70/244 (31.0%)	36/101 (36.4%)	+	+	N.R.	-	+	-	-	-
Polyhydramnios	4/11 (36.4%)	12/53 (23%)	17/37 (46%)	71/226 (31.4%)	57/244 (27.3%)	38/101 (38.4%)	+	+	N.R.	+	+	+	+	+
Oligohydramnios	N.R.	N.R.	N.R.	N.R.	16 (7.7%)	N.R.	-	-	-	-	-	-	-	-
Cryptorchidism	N.R.	N.R.	N.R.	116/120M (96.7%)	118/123M (95.9%)	47/54M (87%)	F	F	+	+	+	+	+	+
Abnormal extremities	1/11 (9.1%)	N.R.	N.R.	58/226 (25.7%)	N.R.	N.R.	+	+	+	-	+	-	-	-
Enlarged lateral vetricle	N.R.	N.R.	N.R.	N.R.	N.R.	2/69 (2.9%)	N.R.	+	+	N.R.	N.R.	N.R.	-	-
Non-reactive CGT pattern	N.R.	N.R.	N.R.	N.R.	N.R.	N.R.	N.R.	N.R.	N.R.	+	-	-	+	-
Fetal cardiac rhabdmyoma	N.R.	N.R.	N.R.	N.R.	N.R.	N.R.	N.R.	N.R.	N.R.	-	N.R.	+	-	-
Small kidneys	N.R.	N.R.	N.R.	N.R.	N.R.	N.R.	N.R.	N.R.	N.R.	N.R.	N.R.	N.R.	N.R.	+
Large BPD	N.R.	N.R.	N.R.	N.R.	N.R.	N.R.	N.R.	NR	N.R.	N.R.	N.R.	N.R.	+	-
Hydronephrosis	N.R.	N.R.	N.R.	N.R.	N.R.	1/69	N.R.	N.R.	N.R.	N.R.	N.R.	N.R.	-	-
SUA	N.R.	N.R.	N.R.	N.R.	N.R.	N.R.	-	-	-	-	-	-	-	+

Abbreviations: DFM, decreased fetal movements; H/A, head/abdomen circumferences; AC, abdominal circumference; SUA, single umbilical artery; BPD, biparietal diameter; N.R., not reported; F, female; M, male; +, feature present; -, feature absent.

## Data Availability

The authors declare that the data for this research are available from the correspondence authors upon reasonable request.

## Abbreviations

The following abbreviations are used in this manuscript:

PWS	Prader–Willi syndrome
MS-MLPA	Methylation-specific multiplex ligation-dependent probe amplification
NIPS	Noninvasive prenatal screening
EFW	Estimated fetal weight
AC	Abdominal circumference
SUA	Single umbilical artery
AFI	Amniotic fluid index
FGR	Fetal growth restriction
CTG	Cardiotocography
UPD	Uniparental disomy

## References

[1] Prader A. (1956). Ein syndrom von adipositas, kleinwuchs, kryptorchismus und oligophrenie nach myatonieartigem zustand im neugeborenenalter. Schweiz. Med. Wochenschr..

[2] Tsai J.H., Scheimann A.O., McCandless S.E., Strong T.V., Bridges J.F.P. (2018). Caregiver priorities for endpoints to evaluate treatments for Prader-Willi syndrome: A best-worst scaling. J. Med. Econ..

[3] Whitman B.Y. (2024). Prader-Willi Syndrome: The More We Know, the Less We Know. Mo. Med..

[4] Butler M.G. (2023). Prader-Willi Syndrome and Chromosome 15q11.2 BP1-BP2 Region: A Review. Int. J. Mol. Sci..

[5] Butler M.G., Miller J.L., Forster J.L. (2019). Prader-Willi Syndrome—Clinical Genetics, Diagnosis and Treatment Approaches: An Update. Curr. Pediatr. Rev..

[6] Mahmoud R., Kimonis V., Butler M.G. (2023). Clinical Trials in Prader-Willi Syndrome: A Review. Int. J. Mol. Sci..

[7] Driscoll D.J., Miller J.L., Cassidy S.B., Adam M.P., Feldman J., Mirzaa G.M., Pagon R.A., Wallace S.E., Amemiya A. (1993). Prader-Willi Syndrome. GeneReviews(®).

[8] Tauber M., Hoybye C. (2021). Endocrine disorders in Prader-Willi syndrome: A model to understand and treat hypothalamic dysfunction. Lancet Diabetes Endocrinol..

[9] Angulo M.A., Butler M.G., Cataletto M.E. (2015). Prader-Willi syndrome: A review of clinical, genetic, and endocrine findings. J. Endocrinol. Investig..

[10] Bravo J.P., Pérez P.D., Canals Cifuentes A. (2021). Nutritional phases of Prader-Willi syndrome. Andes Pediatr..

[11] Höybye C., Tauber M. (2022). Approach to the Patient With Prader-Willi Syndrome. J. Clin. Endocrinol. Metab..

[12] Shubina J., Barkov I.Y., Stupko O.K., Kuznetsova M.V., Goltsov A.Y., Kochetkova T.O., Trofimov D.Y., Sukhikh G.T. (2020). Prenatal diagnosis of Prader-Willi syndrome due to uniparental disomy with NIPS: Case report and literature review. Mol. Genet. Genom. Med..

[13] Hong D.K., Park J.E., Kang K.M., Shim S.H., Shim S.H., Han Y.J., Cho H.Y., Cha D.H. (2023). Prenatal Diagnosis of Uniparental Disomy in Cases of Rare Autosomal Trisomies Detected Using Noninvasive Prenatal Test: A Case of Prader-Willi Syndrome. Diagnostics.

[14] Hiroi H., Kozuma S., Hayashi N., Unno N., Fujii T., Tsutsumi O., Okai T., Taketani Y. (2000). A fetus with Prader-Willi syndrome showing normal diurnal rhythm and abnormal ultradian rhythm on heart rate monitoring. Fetal Diagn. Ther..

[15] Fong B.F., De Vries J.I. (2003). Obstetric aspects of the Prader-Willi syndrome. Ultrasound Obstet. Gynecol..

[16] Geysenbergh B., De Catte L., Vogels A. (2011). Can fetal ultrasound result in prenatal diagnosis of Prader-Willi syndrome?. Genet. Couns..

[17] Fermin Gutierrez M.A., Daley S.F., Mendez M.D. (2025). Prader-Willi Syndrome. StatPearls.

[18] Oto Y., Murakami N., Imatani K., Inoue T., Itabashi H., Shiraishi M., Nitta A., Matsubara K., Kobayashi S., Ihara H. (2023). Perinatal and neonatal characteristics of Prader-Willi syndrome in Japan. Pediatr. Int..

[19] Dong G.Q., Su Y.Y., Qiu X.Y., Lu X.Y., Li J.X., Huang M., Luo X.P. (2020). Clinical screening and genetic diagnosis for Prader-Willi syndrome. Zhongguo Dang Dai Er Ke Za Zhi.

[20] Chitty L.S., Altman D.G. (2003). Charts of fetal size: Kidney and renal pelvis measurements. Prenat. Diagn..

[21] Koirla S., Adhikari K., Devkota K. (2023). Ultrasound Measurement of Fetal Kidney Length in Normal Pregnancy and its Correlation with Gestational Age. J. Nepal. Health Res. Counc..

[22] Choudhary A., Sibia P., Kaur S., Gupta S., Gambhir P., Kaur R. (2024). Evaluation of fetal kidney length as a marker for fetal biometry. Arch. Gynecol. Obstet..

[23] de Vries J.I., Visser G.H., Prechtl H.F. (1982). The emergence of fetal behaviour. I. Qualitative aspects. Early Hum. Dev..

[24] Hadlock F.P., Harrist R.B., Martinez-Poyer J. (1991). In utero analysis of fetal growth: A sonographic weight standard. Radiology.

[25] Leung T.N., Pang M.W., Daljit S.S., Leung T.Y., Poon C.F., Wong S.M., Lau T.K. (2008). Fetal biometry in ethnic Chinese: Biparietal diameter, head circumference, abdominal circumference and femur length. Ultrasound Obstet. Gynecol..

[26] Srebnik N., Gross Even-Zohar N., Salama A., Sela H.Y., Hirsch H.J., Gross-Tsur V., Eldar-Geva T. (2020). Recognizing the unique prenatal phenotype of Prader-Willi Syndrome (PWS) indicates the need for a diagnostic methylation test. Prenat. Diagn..

[27] Bar C., Diene G., Molinas C., Bieth E., Casper C., Tauber M. (2017). Early diagnosis and care is achieved but should be improved in infants with Prader-Willi syndrome. Orphanet J. Rare Dis..

[28] Gross N., Rabinowitz R., Gross-Tsur V., Hirsch H.J., Eldar-Geva T. (2015). Prader-Willi syndrome can be diagnosed prenatally. Am. J. Med. Genet. A.

[29] Zhou Y., Ma M.S., Li G.Y., Zhang Z.J., Ding J., Xu Y.W., Qiu Z.Q., Song H.M. (2021). Analysis of the clinical perinatal characteristics of 226 patients with Prader-Willi syndrome in China. Zhonghua Er Ke Za Zhi.

[30] Grootjen L.N., Uyl N.E.M., van Beijsterveldt I., Damen L., Kerkhof G.F., Hokken-Koelega A.C.S. (2022). Prenatal and Neonatal Characteristics of Children with Prader-Willi Syndrome. J. Clin. Med..

[31] Murata T., Fukuda T., Kanno A., Kyozuka H., Yamaguchi A., Shimizu H., Watanabe T., Fujimori K. (2020). Polyhydramnios and abnormal foetal heart rate patterns in a foetus with Prader-Willi syndrome: A case report. Case Rep. Women’s Health.

[32] Dong Y., Liu S., Li J., Li J., Chen Q., Luo J., Li C., Li H., Qi H., Li R. (2019). Possibility of early diagnosis in a fetus affected by Prader-Willi syndrome with maternal hetero-UPD15: A lesson to be learned. Mol. Med. Rep..

[33] Traisrisilp K., Sirikunalai P., Sirilert S., Chareonsirisuthigul T., Tongsong T. (2022). Cardiac rhabdomyoma as a possible new prenatal sonographic feature of Prader-Willi syndrome. J. Obstet. Gynaecol. Res..

[34] Bigi N., Faure J.M., Coubes C., Puechberty J., Lefort G., Sarda P., Blanchet P. (2008). Prader-Willi syndrome: Is there a recognizable fetal phenotype?. Prenat. Diagn..

[35] L’Herminé A.C., Aboura A., Brisset S., Cuisset L., Castaigne V., Labrune P., Frydman R., Tachdjian G. (2003). Fetal phenotype of Prader-Willi syndrome due to maternal disomy for chromosome 15. Prenat. Diagn..

[36] Salles J., Eddiry S., Lacassagne E., Laurier V., Molinas C., Bieth É., Franchitto N., Salles J.P., Tauber M. (2021). Patients with PWS and related syndromes display differentially methylated regions involved in neurodevelopmental and nutritional trajectory. Clin. Epigenet..

[37] Schwartz L., Caixàs A., Dimitropoulos A., Dykens E., Duis J., Einfeld S., Gallagher L., Holland A., Rice L., Roof E. (2021). Behavioral features in Prader-Willi syndrome (PWS): Consensus paper from the International PWS Clinical Trial Consortium. J. Neurodev. Disord..

[38] Muscogiuri G., Barrea L., Faggiano F., Maiorino M.I., Parrillo M., Pugliese G., Ruggeri R.M., Scarano E., Savastano S., Colao A. (2021). Obesity in Prader-Willi syndrome: Physiopathological mechanisms, nutritional and pharmacological approaches. J. Endocrinol. Investig..

[39] Amaro A.S., Rubin D.A., Teixeira M., Ferreira A.J., Rodrigues G.M., Carreiro L.R.R. (2022). Health Problems in Individuals With PWS Are Associated With Lower Quality of Life for Their Parents: A Snapshot in the Brazilian Population. Front. Pediatr..

[40] Itani R., Gillett E.S., Perez I.A. (2023). Sleep Consequences of Prader-Willi Syndrome. Curr. Neurol. Neurosci. Rep..

[41] Duis J. (2023). Prader-Willi syndrome: An update. Curr. Opin. Pulm. Med..

[42] McQuivey K.S., Chung A.S., Jones M.R., Makovicka J.L., Christopher Z.K., Brinkman J.C., Belthur M. (2022). Hospital outcomes in pediatric patients with Prader-Willi syndrome (PWS) undergoing orthopedic surgery: A 12-year analysis of national trends in surgical management and inpatient hospital outcomes. J. Orthop. Sci..

[43] Kusz M.J., Gawlik A.M. (2022). Adrenal insufficiency in patients with Prader-Willi syndrome. Front. Endocrinol..

[44] Giménez-Palop O., Romero A., Casamitjana L., Pareja R., Rigla M., Caixàs A. (2024). Effect of semaglutide on weight loss and glycaemic control in patients with Prader-Willi Syndrome and type 2 diabetes. Endocrinol. Diabetes Nutr..

[45] Grootjen L.N., Trueba-Timmermans D.J., Damen L., Mahabier E.F., Kerkhof G.F., Hokken-Koelega A.C.S. (2022). Long-Term Growth Hormone Treatment of Children with PWS: The Earlier the Start, the Better the Outcomes?. J. Clin. Med..

[46] Salvatoni A., Nosetti L., Salvatore S., Agosti M. (2021). Benefits of multidisciplinary care in Prader-Willi syndrome. Expert Rev. Endocrinol. Metab..

[47] Ahakoud M., Daha Belghiti H., Nedbour A., Bouramtane A., Chaouki S., Bouguenouch L., Ouldim K. (2023). The Diagnosis and Genetic Mechanisms of Prader-Willi Syndrome: Findings From a Moroccan Population Study. Cureus.

[48] Erhardt É., Molnár D. (2022). Prader-Willi Syndrome: Possibilities of Weight Gain Prevention and Treatment. Nutrients.

[49] Godler D.E., Singh D., Butler M.G. (2025). Genetics of Prader-Willi and Angelman syndromes: 2024 update. Curr. Opin. Psychiatry.

[50] Ma V.K., Mao R., Toth J.N., Fulmer M.L., Egense A.S., Shankar S.P. (2023). Prader-Willi and Angelman Syndromes: Mechanisms and Management. Appl. Clin. Genet..

[51] Salmeri N., Carbone I.F., Cavoretto P.I., Farina A., Morano D. (2022). Epigenetics Beyond Fetal Growth Restriction: A Comprehensive Overview. Mol. Diagn. Ther..

